# Functional Listening Difficulties in Children With ADHD: Associations With Language Abilities

**DOI:** 10.1002/brb3.71763

**Published:** 2026-10-09

**Authors:** Merve Nur SARIYER TEMELLİ, Merve İKİZ BOZSOY, Saniye Tülin FİDAN

**Affiliations:** ^1^ Speech and Language Therapy Department Faculty of Health Sciences Anadolu University Eskişehir Türkiye; ^2^ Child and Adolescent Psychiatry Department Faculty of Medicine Osmangazi University Eskişehir Türkiye

**Keywords:** attention‐deficit/hyperactivity disorder (ADHD), communication outcomes, functional listening, language abilities, listening difficulties

## Abstract

**Objective:**

Listening difficulties are commonly reported in children with attention‐deficit/hyperactivity disorder (ADHD) and are often interpreted as manifestations of inattention. However, it remains unclear whether functional listening difficulties are related to broader language abilities or whether they differ across ADHD subtypes. The present study examined the association between functional listening difficulties and language abilities in children with ADHD and explored whether these relationships vary between predominantly inattentive and combined subtypes.

**Methods:**

This cross‐sectional observational study included 125 children diagnosed with ADHD, categorized into predominantly inattentive (*n* = 32) and combined (*n* = 93) ADHD subtypes. Functional listening was assessed using the Children's Home Inventory for Listening Difficulties (C.H.I.L.D.), with higher scores indicating better functional listening, and language abilities were assessed using the Turkish version of the Test of Early Language Development (TELD‐3:T). Between‐group differences were examined using Mann–Whitney *U* tests. The primary analysis was a hierarchical linear regression examining the adjusted association between total C.H.I.L.D. score and language performance, controlling for age, gender, and ADHD subtype. A C.H.I.L.D.‐by‐ADHD‐subtype interaction was examined in a secondary analysis.

**Results:**

There was no statistical evidence of a difference between ADHD subtypes in C.H.I.L.D. or language scores (all *p* > 0.05). In the adjusted regression, higher total C.H.I.L.D. scores were associated with better language performance (*B* = 6.66, *β* = 0.594, *p* < 0.001, 95% CI [4.996, 8.315]). Exploratory correlations between total C.H.I.L.D. and language scores were positive in the predominantly inattentive type (*r* = 0.72), combined type (*r* = 0.56), and total sample (*r* = 0.61; all *p* < 0.001). The C.H.I.L.D.‐by‐ADHD‐subtype interaction was not significant (*B* = 0.63, *β* = 0.031, *p* = 0.72, 95% CI [−2.919, 4.177]).

**Conclusions:**

Better functional listening was associated with stronger language performance in children with ADHD. There was no statistical evidence that this association differed between ADHD subtypes. These findings highlight the relevance of considering both functional listening and language performance in the clinical assessment of children with ADHD.

## 1 Introduction

Listening plays a central role in children's everyday communication and learning. Effective listening requires the ability to attend to, process, and interpret spoken information in dynamic real‐world environments. In contrast to laboratory‐based listening comprehension tasks that primarily evaluate linguistic understanding, functional listening refers to how effectively individuals manage auditory information in everyday contexts. Functional listening reflects the interaction of multiple processes, including auditory perception, attentional regulation, executive control, and language processing. In clinical settings, caregiver‐reported listening difficulties are frequently used to capture children's listening performance across real‐world environments such as classrooms, social interactions, and noisy situations. Importantly, functional listening should not be considered equivalent to language ability. While language assessments evaluate linguistic knowledge and processing capacity, functional listening reflects how effectively individuals apply these abilities in everyday auditory environments that place demands on attention and executive control.

Attention‐deficit/hyperactivity disorder (ADHD) is a neurodevelopmental condition characterized by persistent patterns of inattention and/or hyperactivity–impulsivity that interfere with functioning across settings (American Psychiatric Association [APA] 2022). Contemporary frameworks conceptualize ADHD as involving alterations in distributed neural systems responsible for executive control and attentional regulation (Faraone and Larsson 2019). Neurodevelopmental studies indicate atypical maturation within frontostriatal and frontoparietal networks in individuals with ADHD, including differences in the prefrontal cortex involved in working memory, inhibitory control, and top‐down attentional regulation (Cortese et al. 2012; Shaw et al. 2007). Consistent with these findings, meta‐analytic research has demonstrated significant impairments in working memory and inhibitory control among children with ADHD (Martinussen et al. 2005; Willcutt et al. 2005). These executive vulnerabilities may affect a range of cognitively demanding activities, including listening and spoken language processing.

Listening comprehension requires the rapid integration of phonological, lexical, and syntactic information while maintaining spoken input in working memory (Baddeley 2003; Just and Carpenter 1992). Successful listening also depends on attentional control mechanisms that allow individuals to sustain focus on relevant speech while suppressing competing auditory stimuli. Because spoken language unfolds rapidly and cannot be revisited in the same way as written text, efficient coordination between attention, working memory, and linguistic processing is essential for effective comprehension. Difficulties in attentional regulation or executive control may, therefore, interfere with real‐time language processing and lead to challenges in everyday listening situations.

These mechanisms are particularly relevant for children with ADHD. Emerging evidence indicates that children with ADHD demonstrate reduced spoken‐word recognition accuracy and slower processing efficiency in challenging listening conditions, such as environments with competing speech (Lemel et al. 2023). Neuroimaging studies further suggest atypical connectivity between auditory cortices and frontoparietal executive networks during listening tasks, indicating potential difficulties in coordinating speech perception with top‐down attentional control. Together, these findings suggest that listening difficulties in ADHD may reflect broader interactions between attentional regulation and language processing systems.

Despite increasing recognition of these interactions, empirical research examining listening abilities in ADHD remains limited. Many studies have investigated language abilities, academic outcomes, or attentional symptoms separately, making it difficult to determine how listening difficulties relate to broader language functioning in this population. Furthermore, listening outcomes are often measured using tasks that primarily assess linguistic comprehension rather than functional listening performance in real‐world environments. This distinction is important because listening in everyday contexts requires children to manage auditory information while simultaneously coping with attentional demands, environmental distractions, and competing stimuli.

Another unresolved issue concerns whether listening difficulties differ across ADHD subtypes. ADHD is typically classified into predominantly inattentive, predominantly hyperactive‐impulsive, and combined subtypes based on symptom patterns (APA 2022). However, neurocognitive evidence suggests that executive control and attentional regulation systems are affected across subtypes (Cortese et al. 2012; Faraone and Larsson 2019). Consequently, functional listening difficulties may not necessarily differ between ADHD subtypes but may instead reflect shared cognitive mechanisms influencing language processing and attentional control.

Understanding how functional listening relates to language abilities within ADHD populations may have important implications for clinical assessment and intervention. Listening complaints in children with ADHD are often interpreted primarily as manifestations of behavioral inattention. However, such difficulties may also reflect vulnerabilities in language processing that interact with attentional regulation during everyday communication. Examining the relationship between listening and language abilities may, therefore, provide insight into how attentional and linguistic mechanisms jointly contribute to communication challenges in ADHD.

Accordingly, the present study aimed to compare functional listening difficulties and language performance between children with predominantly inattentive and combined ADHD subtypes, and to examine associations between listening difficulties and language abilities within a clinical ADHD sample. Based on previous evidence indicating that executive dysfunction occurs across ADHD subtypes (Martinussen et al. 2005; Willcutt et al. 2005; Sarıyer et al. 2023), it was hypothesized that listening and language scores would not significantly differ between subtypes, but that functional listening difficulties would be associated with language performance. Clarifying these relationships may contribute to a better understanding of how attentional regulation and language processing interact in ADHD and may inform multidisciplinary approaches to the assessment and management of communication difficulties in affected children.

## 2 Material and Methods

### 2.1 Study Design

This cross‐sectional observational study was conducted at the child and adolescent psychiatry outpatient clinic of Osmangazi University between June 2025 and December 2025. Participants were recruited using a convenience sampling method from children diagnosed with ADHD who attended the outpatient clinic during the study period. A total of 125 children with an established ADHD diagnosis met the eligibility criteria and were enrolled in the study. No participants were excluded after eligibility assessment, and no participants withdrew from the study. Therefore, all 125 enrolled participants were included in the final analyses. No missing data were identified for the variables included in the analyses.

### 2.2 Participants

A total of 125 children diagnosed with ADHD participated in the study. Participants were categorized into predominantly inattentive (AD; *n* = 32) and combined (CT; *n* = 93) subtypes. The CT group included 18 females (19.4%) and 75 males (80.6%), whereas the AD group included 29 females (90.6%) and 3 males (9.4%). The median age was 6.20 years (range: 4.10–7.90) in the CT group and 6.35 years (range: 4.30–7.80) in the AD group, with no significant age difference between groups (*p* = 0.29).

ADHD diagnoses were established by a child and adolescent psychiatrist according to DSM‐5 criteria. Children with comorbid neurodevelopmental or psychiatric disorders, hearing impairment, neurological conditions, or IQ below 85 were excluded.

Because of the marked difference in sex distribution between the ADHD subtypes, sex was included as a covariate in the primary regression analysis. Other potentially relevant clinical and sociodemographic variables, including ADHD symptom severity, medication status, caregiver sex and relationship to the child, caregiver educational level, and socioeconomic background, were not systematically collected and, therefore, could not be included in the statistical models.

### 2.3 Data Collection Process

Participants were recruited from a child and adolescent psychiatry outpatient clinic. Functional listening difficulties were assessed using the Children's Home Inventory for Listening Difficulties (C.H.I.L.D.), and language abilities were evaluated with the Test of Early Language Development (TELD‐3:T). Caregivers completed the C.H.I.L.D. questionnaire, while language assessments were administered directly to children. Data collection was conducted by a multidisciplinary team including a speech‐language therapist, an audiologist, and a child psychiatrist, in accordance with the Declaration of Helsinki.

This study was approved by the Anadolu University Health Sciences Scientific Research and Publication Ethics Committee (Protocol No: 889468; Approval Date: May 28, 2025). Written informed consent was obtained from the parents or legal guardians of all participating children prior to data collection. All procedures were conducted in accordance with the ethical standards of the institutional research committee and the Declaration of Helsinki.

### 2.4 Measurements

#### 2.4.1 Children's Home Inventory for Listening Difficulties Questionnaire

The C.H.I.L.D. questionnaire (Anderson and Smaldino 2000) assesses listening difficulties in everyday situations. The family member version (C.H.I.L.D.–FM), designed for children aged 3–12 years, was used in the present study. The Turkish version has demonstrated strong validity and reliability (Yıldırım Gökay et al. 2024). The scale includes subdomains assessing listening in quiet, noise, distance, social settings, and media contexts.

#### 2.4.2 Test of Early Language Development

The Turkish version of the TELD‐3:T was used to assess participants’ receptive and expressive language development. The original TELD‐3 was developed by Hresko et al. (1999). The normative sample for the Turkish validity and reliability study consisted of 1200 typically developing children aged between 18 months and 8 years (Topbaş&Güven 2011). The findings demonstrated that the TELD‐3:T has strong and statistically significant validity and reliability. Furthermore, the test was shown to accurately assess receptive and expressive language skills and to identify children with language delays and language disorders.

The TELD‐3:T combines verbal instructions with visual stimuli, such as objects or pictures, to which the child is expected to respond. In the current study, TELD‐3:T results were presented as spoken language standard scores. The overall spoken language development score was calculated by combining the receptive and expressive language scores.

### 2.5 Statistical Analysis

Statistical analyses were performed using SPSS version 23.0. The normality of continuous variables was assessed using histograms, probability plots, and the Kolmogorov–Smirnov and Shapiro–Wilk tests. Because the variables were not normally distributed, continuous variables were summarized using medians and minimum–maximum values, and categorical variables were summarized using frequencies and percentages.

The primary analysis was defined as an adjusted linear regression examining the association between total C.H.I.L.D. score and TELD‐3 language score in the full sample. Age, gender, and ADHD subtype were entered as covariates in the first step, followed by the total C.H.I.L.D. score in the second step. To examine whether the association between total C.H.I.L.D. score and language performance differed according to ADHD subtype, a C.H.I.L.D.‐by‐ADHD‐subtype interaction term was entered in a third step. The total C.H.I.L.D. score was mean‐centered before the construction of the interaction term.

Between‐group differences in C.H.I.L.D. and TELD‐3 scores were examined using the Mann–Whitney *U* test. Spearman rank correlations were used for ADHD‐subtype‐specific and subscale analyses, which were considered exploratory. Holm's sequential procedure was applied to the five C.H.I.L.D. subscale correlations within each analysis group to account for multiple comparisons. The total C.H.I.L.D. score was considered the primary predictor and was not included in the subscale multiplicity adjustment. Statistical significance was set at *p* < 0.05.

## 3 Results

The median total C.H.I.L.D. score was 4.26 (1.66–7.46) in the CT group and 4.57 (2.73–7.26) in the AD group. There was no statistically significant difference between the groups in the C.H.I.L.D. total scores (*p* = 0.17). In addition, there were no statistically significant differences between the groups in the quiet (*p* = 0.69), noisy (*p* = 0.62), distance (*p* = 0.17), social (*p* = 0.40), and media (*p* = 0.92) subtest scores (Table 1).

**TABLE 1:** Comparison analysis of measurement results.

	CT group (*n* = 93)		AD group (*n* = 32)		
	Median (IQR)	Min–max	Median (IQR)	Min–max	*p*‐value^a^
**C.H.I.L.D quiet**	4 (1)	1–8	4.25 (1.50)	2.25–7	0.69
** Noisy**	4 (1.75)	1–7.25	4 (2)	2.25–7.75	0.62
** Distance**	4 (3)	1–7.66	5 (2.16)	3–8	0.17
** Social**	4 (1.67)	1–8	4.17 (1.67)	3–8	0.40
** Media**	4 (1)	1–8	4.5 (1.50)	3–7	0.92
** Total**	4.26 (1.07)	1.66–7.46	4.57 (1.4)	2.73–7.26	0.17
**TELD‐3 language score**	79 (15)	60–125	80 (18)	47–103	0.49

^a^ Mann–Whitney *U* test.

The median language score was 79 (60–125) in the CT group and 80 (47–103) in the AD group. There was no statistically significant difference in language scores between the groups (*p* = 0.49) (Table 1).

In the primary hierarchical regression analysis, age, gender, and ADHD subtype were entered in the first step, explaining 5.9% of the variance in language scores (*R* ^2^ = 0.059, adjusted *R* ^2^ = 0.035, ΔF(3,121) = 2.508, *p* = 0.06). Addition of the total C.H.I.L.D. score in the second step significantly improved the model (Δ*R* ^2^ = 0.324, ΔF(1,120) = 63.07, *p* < 0.001), resulting in an *R* ^2^ of 0.383 (adjusted *R* ^2^ = 0.362). In Model 2, total C.H.I.L.D. score was significantly associated with language score (*B* = 6.66, SE = 0.84, *β* = 0.594, *p* < 0.001, 95% CI [4.996, 8.315]), whereas age, gender, and ADHD subtype were not statistically significant (all *p* > 0.05).

In the secondary interaction analysis, the C.H.I.L.D.‐by‐ADHD subtype interaction was not statistically significant (*B* = 0.63, SE = 1.79, *β* = 0.031, *p* = 0.72, 95% CI [−2.919, 4.177]). The addition of the interaction term explained virtually no additional variance (Δ*R* ^2^ = 0.001, ΔF(1,119) = 0.123, *p* = 0.72). Thus, there was no statistical evidence that the association between total C.H.I.L.D. score and language performance differed between the combined and predominantly inattentive ADHD subtypes (Table 2).

**TABLE 2:** Hierarchical linear regression analysis predicting TELD‐3 language score.

Regression step	Variable	*B*	SE	*β*	95% CI for *B*	*p*	*R*	*R* ^2^	Adjusted *R* ^2^	Δ*R* ^2^	ΔF	*p* for ΔF
**Step 1**	Constant	72.43	7.39	—	57.800–87.072	< 0.001	0.242	0.059	0.035	0.059	2.508	0.06
	Age	1.825	1.12	0.144	−0.408–4.058	0.10						
	Gender	−5.92	2.76	−0.247	−11.401–−0.455	0.03						
	ADHD subtype	−4.12	3.07	−0.155	−10.208–1.951	0.18						
**Step 2**	Constant	76.42	6.03	—	64.486–88.369	< 0.001	0.619	0.383	0.362	0.324	63.07	< 0.001
	Age	0.74	0.92	0.059	−1.091–2.580	0.42						
	Gender	−2.07	2.29	−0.086	−6.625–2.480	0.36						
	ADHD subtype	−3.20	2.49	−0.120	−8.154–1.743	0.20						
	**Total C.H.I.L.D. score**	**6.66**	**0.84**	**0.594**	**4.996–8.315**	**< 0.001**						
**Step 3**	Constant	76.59	6.07	—	64.571–88.616	< 0.001	0.619	0.384	0.358	0.001	0.123	0.72
	Age	0.73	0.93	0.058	−1.111–2.577	0.43						
	Gender	−2.20	2.34	−0.092	−6.843–2.425	0.34						
	ADHD subtype	−3.38	2.55	−0.127	−8.450–1.684	0.18						
	Total C.H.I.L.D. score	6.45	1.01	0.577	4.441–8.469	< 0.001						
	**C.H.I.L.D. × ADHD subtype**	**0.63**	**1.79**	**0.031**	**−2.919–4.177**	**0.72**						

*Note*: Step 1 included age, gender, and ADHD subtype. Step 2 added total C.H.I.L.D. score and represents the primary adjusted analysis. Step 3 added the C.H.I.L.D.‐by‐ADHD‐subtype interaction term to test whether the association between total C.H.I.L.D. score and TELD‐3 language score differed by ADHD subtype. B = unstandardized regression coefficient; *β* = standardized regression coefficient; *R* = multiple correlation coefficient; *R* ^2^ = coefficient of determination; Δ*R* ^2^ = change in *R* ^2^; ΔF = change in F statistic.

Abbreviations: CI = confidence interval; SE = standard error.

Exploratory Spearman correlation analyses showed positive associations between language scores and all C.H.I.L.D. subscales and the total score in the overall sample and within both ADHD subtypes. For the total C.H.I.L.D. score, the correlation was moderate in the CT group (*ρ* = 0.56, *p* < 0.001), strong in the AD group (*ρ* = 0.72, *p* < 0.001), and strong in the total sample (*ρ* = 0.61, *p* < 0.001). However, the nonsignificant C.H.I.L.D.‐by‐ADHD‐subtype interaction provided no statistical evidence that the strength of this association differed between ADHD subtypes.

The magnitude of the subscale associations ranged from weak to strong. In particular, the social‐domain association was weak in the CT group (*ρ* = 0.24, Holm‐adjusted *p* = 0.020) and in the total sample (*ρ* = 0.30, Holm‐adjusted *p* < 0.001), despite statistical significance. Other subscale associations were moderate to strong. Detailed correlation coefficients and Holm‐adjusted *p*‐values are presented in Table 3.

**TABLE 3:** Correlation analysis of measurement results.

Outcome	Group	Quiet		Noisy		Distance		Social		Media		Total	
		*p*	*r*	*p*	*r*	*p*	*r*	*p*	*r*	*p*	*r*	*p*	*r*
**TELD‐3 score**	CT group	< 00.001	0.59^b^	< 0.001	0.41^b^	< 0.001	0.48^b^	0.020	0.24^a^	< 0.001	0.54^b^	< 0.001	0.56^b^
	AD group	< 0.001	0.77^c^	0.003	0.54^b^	< 0.001	0.65^c^	0.015	0.42^b^	< 0.001	0.74^c^	< 0.001	0.72^c^
	Total sample	< 0.001	0.64^c^	< 0.001	0.43^b^	< 0.001	0.52^b^	<0.001	0.30^a^	< 0.001	0.59^b^	< 0.001	0.61^c^

*Note*: Values are presented as *p*‐value/Spearman's r. Holm's sequential procedure was applied to adjust *p*‐values for the five exploratory C.H.I.L.D. subscale correlations within each group. Total C.H.I.L.D. score correlations were not included in the multiplicity adjustment because the total score was the primary predictor.

^a^ Weak correlation.

^b^ moderate correlation.

^c^ strong correlation.

## 4 Discussion

The present study examined functional listening difficulties and language performance in children with predominantly inattentive and combined subtypes of ADHD and explored the relationship between listening and language abilities within this clinical population. The findings showed no statistically significant differences between ADHD subtypes in functional listening or language performance. However, positive associations were observed between functional listening and language performance in the total sample and within each ADHD subtype. These findings indicate that functional listening was associated with language performance in the present ADHD sample, while the study did not provide statistical evidence of a subtype‐level difference in these outcomes.

The absence of statistically significant between‐subtype differences should be interpreted cautiously. Both ADHD subtypes included in the present study are characterized by inattention, whereas the combined type additionally includes clinically significant hyperactive‐impulsive symptoms. Therefore, the present comparison can identify whether functional listening and language performance differ at the level of ADHD subtypes, but it cannot isolate the independent contribution of hyperactive‐impulsive symptoms to these outcomes. Accordingly, the nonsignificant group comparisons should be interpreted as no statistical evidence of a subtypes‐level difference, rather than as evidence that the two subtypes are equivalent or that they share underlying neurocognitive mechanisms.

Although previous research has reported executive function and attentional difficulties across ADHD subtypes(Cortese et al. 2012; Faraone and Larsson 2019; Martinussen et al. 2005; Willcutt et al. 2005), the present study did not directly measure neurocognitive mechanisms. Therefore, such mechanisms cannot be inferred from the observed nonsignificant subtype‐level differences. Future studies using direct measures of attention, executive functioning, and hyperactivity‐impulsivity are needed to determine whether these factors independently contribute to functional listening and language performance.

This interpretation is consistent with neuroimaging findings showing atypical connectivity within frontoparietal and frontostriatal networks that support executive control and attentional allocation in ADHD (Cortese et al. 2012). These networks play an important role in maintaining attention during speech perception, particularly in challenging listening environments such as background noise or competing speech. Consequently, listening difficulties reported by caregivers may reflect limitations in attentional regulation rather than subtype‐specific behavioral characteristics. From this perspective, functional listening difficulties may reflect the interaction between attentional regulation and language processing mechanisms.

A key finding of the present study was the positive association between functional listening and language performance. This association was observed in the total sample and within both ADHD subtypes, with the adjusted regression analysis indicating that higher C.H.I.L.D. scores were associated with stronger language performance after adjustment for age, sex, and ADHD subtype. However, because of the cross‐sectional observational design, this association should not be interpreted as evidence of a causal relationship. In addition, unmeasured factors, particularly inattentive symptom severity, could not be accounted for in the present analyses.

Listening comprehension requires the real‐time integration of phonological, lexical, and syntactic information while maintaining and updating information in working memory (Baddeley 2003; Just and Carpenter 1992). The observed association is consistent with the possibility that attentional and linguistic processes are related during everyday listening; however, the present data do not allow the independent contribution of these mechanisms to be determined.

Importantly, the association between functional listening and language abilities should not be interpreted as measurement redundancy. Functional listening reflects a multidimensional construct that emerges from the interaction of auditory perception, attentional control, executive functioning, and linguistic processing in everyday listening environments. In contrast, standardized language assessments primarily evaluate linguistic knowledge and processing capacity. The relationship observed in this study therefore likely reflects the interaction between attentional regulation and language processing mechanisms rather than overlap between identical constructs.

These findings have important implications for how listening complaints in children with ADHD are interpreted in clinical and educational contexts. Listening difficulties are often attributed primarily to behavioral inattention, which may lead clinicians and educators to focus mainly on interventions targeting attentional regulation. The present results suggest that listening problems may also reflect vulnerabilities in language processing (Cain and Oakhill 2007; Faraone& Larsson 2019). Accordingly, clinical assessment may benefit from integrating both listening and language evaluations rather than attributing listening difficulties solely to attentional symptoms.

Multidisciplinary approaches may therefore be particularly valuable when evaluating communication challenges in children with ADHD. Collaborative assessment involving child and adolescent psychiatry, audiology, and speech‐language therapy may provide a more comprehensive understanding of how attentional, auditory, and language‐related factors interact to influence everyday communication. Intervention strategies that address both attentional regulation and listening environments—for example, through reducing background noise, supporting structured listening contexts, and adapting instructional strategies—may also contribute to improved communication and academic participation.

Overall, the findings support a more integrated perspective on communication difficulties in ADHD. Rather than reflecting purely behavioral manifestations of inattention, functional listening difficulties may arise from the interaction between attentional regulation and language processing mechanisms during everyday communication. Considering these interacting processes may help clinicians and educators better identify and address the communication needs of children with ADHD.

## 5 Conclusion

The present findings indicate that there was no statistical evidence of a subtype‐level difference in functional listening difficulties or language performance between children with predominantly inattentive and combined ADHD subtypes. Importantly, because both subtypes involve inattention and the combined subtype additionally includes hyperactive‐impulsive symptoms, these findings should not be interpreted as evidence of equivalence between subtypes or as demonstrating shared underlying mechanisms.

Higher C.H.I.L.D. scores, indicating better functional listening, were associated with stronger language performance in the overall ADHD sample after adjustment for age, sex, and ADHD subtype. However, the cross‐sectional design and the absence of detailed measures of inattentive symptom severity and other potential confounders limit causal and mechanistic interpretations.

These findings support the clinical relevance of considering functional listening and language performance together when assessing children with ADHD. Further studies incorporating direct measures of ADHD symptom dimensions, including inattentive and hyperactive‐impulsive symptoms, as well as longitudinal designs, are needed to clarify the factors underlying the association between functional listening and language performance.

### 5.1 Study Limitations

Several limitations should be considered when interpreting the findings. First, the study did not include a typically developing control group. The primary aim, however, was not to compare children with ADHD to typically developing peers, but rather to examine variability within ADHD subtypes and to explore the relationship between functional listening difficulties and language abilities within a clinically diagnosed ADHD sample. Nevertheless, future studies including typically developing comparison groups may help clarify whether the observed listening–language associations are specific to ADHD or reflect broader developmental patterns.

Second, children with the predominantly hyperactive‐impulsive subtype were not represented in the sample. This likely reflects clinical referral patterns, as children with prominent hyperactivity symptoms are often identified earlier due to overt behavioral concerns, whereas inattentive symptoms may lead to later referral associated with academic difficulties. Consequently, the findings cannot be generalized to all ADHD subtypes, and future studies should include all diagnostic subtypes to allow more comprehensive subtype comparisons.

Third, detailed information regarding age at ADHD diagnosis and duration of treatment prior to participation was not consistently available. Variability in diagnostic timing and treatment history may influence language development and functional listening outcomes, potentially contributing to heterogeneity within the sample. Longitudinal research incorporating such clinical variables would help clarify developmental trajectories and treatment effects on communication outcomes.

An additional limitation concerns the marked imbalance in sex distribution between the ADHD subtypes. Although sex was included as a covariate in the primary regression analyses, the predominantly inattentive subtype was composed primarily of females, whereas the combined subtype consisted predominantly of males. Consequently, some observed subtype‐related patterns may partially reflect sex‐related differences rather than ADHD subtype characteristics alone. Future studies should aim to recruit more balanced samples across sex and ADHD subtype categories to better disentangle the potential contributions of these factors.

In addition, several potentially important confounding variables were not systematically collected, including ADHD symptom severity, particularly the severity of inattentive symptoms, medication status, caregiver sex and relationship to the child, caregiver educational level, and socioeconomic background. Therefore, these variables could not be controlled for in the present analyses. Although the primary regression model adjusted for age, sex, and ADHD subtype, the absence of detailed measures of inattentive symptom severity limits the extent to which the observed association between functional listening and language performance can be interpreted as independent of attentional symptom burden. Future studies should incorporate standardized measures of ADHD symptom severity and relevant clinical and sociodemographic covariates to determine whether the listening–language association remains after more comprehensive adjustment.

Finally, listening difficulties were assessed using caregiver‐report questionnaires rather than objective listening performance tasks. Although the instrument demonstrates strong psychometric properties, parental perceptions may be influenced by behavioral characteristics beyond auditory processing itself. Future studies combining caregiver reports with objective listening or auditory processing measures may further clarify mechanisms underlying functional listening difficulties in ADHD.

Overall, these findings suggest that listening complaints in children with ADHD may reflect the interaction between attentional regulation and language processing mechanisms rather than solely behavioral inattention, highlighting the importance of multidisciplinary evaluation approaches.

## Author Contributions

**Merve Nur Sariyer Temelli**: conceptualization, investigation, methodology, Writing – review and editing, Writing – original draft, supervision, resources. **Merve Ikiz Bozsoy**: conceptualization, methodology, writing – original draft, investigation, software, writing – review & editing, formal analysis, data curation, conceptualization, supervision. **Saniye Tülin Fidan**: methodology, investigation, writing – original draft, writing – review and editing, supervision, conceptualization.

## Funding

This work has been supported by Anadolu University Scientific Research Projects Coordination Unit under grant number THD‐2026‐3642.

## Conflicts of Interest

The authors declare no conflicts of interest.

## Data Availability

The data that support the findings of this study are available from the corresponding author upon reasonable request.
